# The Effect of Teacher Caring Behavior and Teacher Praise on Students’ Engagement in EFL Classrooms

**DOI:** 10.3389/fpsyg.2021.746871

**Published:** 2021-09-14

**Authors:** Yadi Sun

**Affiliations:** School of Foreign Languages, Zhongnan University of Economics and Law, Wuhan, China

**Keywords:** teacher caring behavior, teacher praise, students’ engagement, EFL classroom, teacher’s effect on language teaching

## Abstract

The emergent respect for the prominence of engagement in the present education has made it one of the most widespread inquiry issues that it has been regarded as the ultimate target of learning. In the language teaching field, the idea of student activities for learning is intensely rooted in the prevailing standards of effective language learning, which considers language communication and interaction as analytical for language improvement. Moreover, teachers as center of learning process is the most prominent research attention, and teachers play a key role in regulating the education process as well as students’ learning achievement. However, there is an absence of research which have considered teachers’ care and praise among all positive interpersonal behavior and its significant effect on students’ engagement. So, the present review attempts to focus on teacher care and praise, and their effects on student engagement in EFL classrooms. Subsequently, some implications are presented to clarify the practice of teachers, students, teacher educators, and materials developers.

## Introduction

In language learning, some students are not motivated enough, so they lose their primary attentiveness that ultimately results in dropping out and quitting without graduation (Finn and Zimmer, 2012), and lack of their engagement has a remarkable role in this way (Fredricks et al., 2004; Ladd and Dinella, 2009). That is to say, students give up studying English because they become less engaged; hence, they might lose their initial interest gradually, which can result in dropping out.

Student engagement, as a strategic factor of learner achievement in higher education, has been at the center of the attention of directors, experts, and scholars in the previous decade (Kahu and Nelson, 2018). In language learning, some researchers have paid attention to engagement in the classroom and have been of service to this domain to date (Philp and Duchesne, 2016; Oga-Baldwin, 2019). Second language acquisition (SLA) has undertaken a shift toward Positive Psychology (PP; MacIntyre et al., 2019), which has fortified studies on engagement, focusing on some vital PP aspects embedded in the heart of positive language learning (Mercer and Dörnyei, 2020; Wang et al., 2021).

Engagement is two sides of the coin; one side is disengagement that is what learners do to elude attending to learning tasks, while the other side is the way a student is involved in these tasks (Lei et al., 2018). Engagement arises moderately out of “bright side” precursors, such as teacher care which derives from self-determination theory (SDT; Ryan and Deci, 2017). In line with SDT, all learners have a series of three fundamental widespread spiritual needs that is those for autonomy (need to encounter preference and self-authorization in individual’s performance), competence (need to sustain progress and a nous of reflectance in one’s communications with the situation), and relatedness (need to go through deep, approachable, and mutual care within individual’s interactions; Mercer, 2019; Reeve et al., 2019). The fulfillment of these needs throughout language learning regulates the degree to which learners flourish and show adaptive functioning, namely, inherent motivation and engagement (Reeve et al., 2018). The relationships between teacher and student, which can be associated with learners’ basic emotional needs, are among the levels of encouragement on learner improvement (Froiland et al., 2019; Xie and Derakhshan, 2021) and are the outcomes of a continuing interaction between the educator and the learner’s features (Sabol and Pianta, 2012). Relatedness can envisage learner behavioral, emotional, and agentic engagement since good teacher-student relations can boost learners’ participation (behavioral engagement), nurture students’ optimistic outlooks toward the course and its tasks (emotional engagement), provide learners the self-confidence to work on difficult actions (cognitive engagement), and boost learners to speak out concerning their education requirements (agentic engagement; Ruzek et al., 2016; Vollet et al., 2017).

Student engagement is noteworthy since it can envisage learners’ success or academic advancement (Ladd and Dinella, 2009). Also, student engagement is by some issues, such as teacher support or social experiences that provide teachers with instant feedback and praise on their efforts to inspire learners during the teaching process for assessment determinations (Reeve, 2012). It is maintained that engagement would be the best educational indicator of students’ motivation and its dynamic and flexible features (Appleton et al., 2006) entail that it can be reformed as a result of various intrapersonal and interpersonal ecological elements (Fredricks et al., 2004). Teachers’ interpersonal relationship is the main element in the academic setting that exerts a central effect on students’ engagement (Jiang and Zhang, 2021). Likewise, student engagement is inclined by relative variants, such as learning situations or approaches and techniques utilized by teachers (Fredricks et al., 2019). Some longitudinal research (Jang et al., 2016; Reeve et al., 2018) within the SDT framework indicated that modifications in student engagement are affected by their learning milieu and such relation is mutual; when situating in classroom circumstances, this ecological element is hypothesized as their teachers’ diverse appealing practice. In previous theories, student engagement has been proposed to be mutually a procedure and a consequence (Reschly and Christenson, 2012). It might be perceived as an interpersonal development triggered by reciprocal interpersonal interactions (Pianta et al., 2012), while as a consequence, student engagement refers to what learners do in the process of intervention. Furthermore, it is adjudged as a moderator between learners’ academic settings and student consequences (Appleton et al., 2006).

Moreover, the success of language learning in the classroom depends on the teacher, and it is stated that students’ achievement or failure in learning can rely on the efficacy of the teachers (Luz, 2015). Sarter (2012) declared that human emotions have been brought to light recently. Although among the emotions that have been tackled so far, anxiety, depression, and stress have been the most prominent, during two previous decades, positive emotions, like love, pride, hope, and enjoyment, have been brought into view (MacIntyre and Mercer, 2014), as generated by the advent of PP into second language learning (MacIntyre and Mercer, 2014; MacIntyre et al., 2019; Wang et al., 2021). Correspondingly, there has been growing consideration to the role of emotions in teachers’ lives, and emotion is a crucial element of teaching (Samier and Schmidt, 2009). It has been revealed that most teachers all around the world feel negative in language learning classrooms, so it can be proposed that teachers’ emotions should go beyond emotional issues and should turn into social aspects. Thus, sociology can be considered as an agenda to be aware of the social foundations of teachers’ feelings (Tsang, 2015). Lacking constructive emotions, teachers may not be interested or motivated to develop students’ academic, social, and emotional progress (Day and Qing, 2009). The significant role of positive emotions is assured as it helps to foster students’ emotional powers and wellbeing, and encourages social manners and it organizes the social properties for the students’ achievement in language learning (Zhang et al., 2019). Prosocial behavior is a premeditated action to assist others when it is done intentionally instead of reacting to another’s command or by the expectancy of a reward or reprimand (Grusec et al., 2011). Prosocial manners happen as peers support, care, collaborate, and demonstrate respect for each other, and variations in learners’ prosocial behavior are more receptive to sympathetic interactions and involving students in a caring situation (Cheon et al., 2018).

Furthermore, the relations between teachers and learners and teacher manners can meaningfully influence student engagement (Groves et al., 2015). Through interactions with teachers, the students encounter emotional and attitudinal stability and obtain satisfactory emotional support from their teachers which results in effective learning (Pekrun and Schutz, 2007). As stated by Malaimakuni (2016), effective teachers must have adequate knowledge of the subject matter and in giving their knowledge to students, they routinely should have noble relational communication. Among numerous issues, which provide emotional support for language learners, the relationship between teacher and learner is prominent which is actualized as the most authoritative tool that teachers have, when trying to cultivate a satisfactory learning setting (Strachan, 2020; Li and Yang, 2021; Xie and Derakhshan, 2021). Because the teacher-learner relationship is dominant to the satisfaction of learners’ emotional needs, scholars have emphasized its quality and nature (Pishghadam and Khajavy, 2014). This relationship in the classroom is essential for not only teachers’ progress but also students’ progress (Delos Reyes and Torio, 2020). Therefore, they must cooperate together to build worthwhile learning circumstances in which the teacher motivates the formation of such situations by taking on relational performances that are related to students’ positive involvements (Bolkan et al., 2015).

A positive relationship between the teacher and learners may be thoroughly associated with the passions, particularly the positive ones emphasized in PP, that students may face within the route of language learning (Dewaele et al., 2019; McIntyre et al., 2019). A positive relationship between teacher and learner is acknowledged by empathy, caring, participation, hope, and esteem, and all examples of teacher positive behaviors investigated to date are teacher care, stroke, immediacy, credibility, simplicity, approval, and praise (Frisby, 2019). It is hypothesized that for increasing student engagement, teachers should be friendly, sympathetic, sociable to students’ distinctiveness, support learners’ independence, and be eager about their careers (Frisby, 2019). To this end, teachers can take on varied roles, such as taking care of their conversation, being cautious about feedback to learners, listening to them, providing inquiries to involve learners, and reconsidering classroom management to control relations (Mercer and Dörnyei, 2020). In academic state, the construct of “care” has been broadly investigated and is evolving as an essential element of successful education (Velasquez et al., 2013; Pishghadam et al., 2019). In this situation, caring encompasses presenting emotional provision and venture in the rapport with learners, and it is similar to what individuals do and say in their performances and communications (Davis et al., 2012).

Furthermore, caring has been clarified as those feelings, activities, and thoughts that emanate from an educator’s aspiration to stimulate, help, engage, or motivate their learners (O’Connor, 2008). Concentrating on the mutual nature of this construct, it is claimed that “caring education is the performance that arises from a reciprocal caring relationship between learner and teacher, where learning takes place through modeling, discourse, and approval at the social levels” (Velasquez et al., 2013). Teacher care inspires learner-related capabilities, such as engagement, self-confidence, wellbeing, feeling appreciated, and achievement (Derakhshan et al., 2019; Havik and Westergård, 2020).

In addition, praise has been reflected as providing encouragement, self-confidence, and good teacher-learner relationships and it is believed that in educational psychology, teacher praise is an essential basis of support for effective student presentation and an indispensable and influential part of teaching and it is a noteworthy strategy in engaging student in the route of learning and praising students in the class fosters language students’ learning motivation and behaviors (Guilloteaux and Dörnyei, 2008). Praising is a technique to reward students who involve in echoing good behaviors or accomplishing better presentations to take advantage of praise (Brophy, 2004).

Undoubtedly, the reward of the teacher is a type of gratefulness of the work of learners which can be done through phrasing praise proclamations as a statement of educational response and feedback instead of appraisal and assessment (Brophy, 2004). This is congruent with Deci et al. (2001) who pinpointed that verbal rewards should be explanatory more willingly than regulatory since the regulatory types are inclined to challenging motivation. Verbal praise should embrace gratefulness of the students’ presentation because the teacher is acting two things equivalently; he is gratifying learners for their performance while teaching them how to allocate their determinations to their inherent enthusiasm rather than to extrinsic motivations provided by the teacher (Brophy, 2004), and it is evinced that the use of praise brings about inspiring learners of having a feeling of superiority and self-assurance in their abilities and accomplishments. As Firdaus (2015) stated, the use of praise will be influential if the teachers discern about it well and how to employ it. Moreover, Hodgman (2015) claimed that praise can be positive support toward students’ manners and confronts the students in a challenging setting to be involved in the educational inquiries and decrease the learners’ problems. Teacher praise is frequently acclaimed as behavior management preparation, which is maintained by some studies (Conroy et al., 2008; Epstein et al., 2008).

Taken together the significance of teacher caring behavior and praise as types of positive interpersonal interactions in language education, on the one hand, and learner engagement, on the other hand, it seems required that investigating the rapport between these two constructs has superiority. Furthermore, the rapport between student engagement and teacher caring behavior and teacher praise has not been much investigated in the language education field thus far. Accordingly, the aim of the present study was to bridge this lacuna by investigating the connection between student engagement and teacher praise and caring behavior in the EFL context.

## Student Engagement: Definition and Dimensions

As stated by Dörnyei (2018), student engagement as a whole relates to involvement in educational tasks and activities. More accurately, engagement can be illuminated as the level of a student’s enthusiastic participation in instructional activities (Reeve, 2012), or a person’s extreme participation in an action (Reeve et al., 2004). Concerning the dimensions of learner engagement, Appleton et al. (2006) utilized the *cognitive* and *emotional* modules to measure student engagement. Hart et al. (2011) focused on the three sub-constituents to measure student engagement, namely, *emotional, cognitive*, and *behavioral*, and another component which is *agentic* was added by Reeve (2013).

The behavioral engagement is elucidated as the noticeable educational presentation and sharing activities and tasks which is evaluated through visible educational act containing: student’s positive behavior; participation; attempt to focus on activities; involvement in class negotiations; contribution in educational and co-curricular tasks; persistence; and resiliency, when confronted with challenging actions (Khademi Ashkzari et al., 2018).

The affective or emotional aspect of engagement relates to the cumulative and permanent degrees of emotions encountered by learners and gains the level of desire learners perceive toward the tertiary knowledge (Bowden, 2013), and this type of engagement evinced through intensified levels of positive emotions during activities, which may be presented through pleasure, superiority, enjoyment, eagerness, and interest (Klem and Connell, 2004). Students who are passionately involved in academic activities are capable of detecting the objective of the tasks and social communications (Schaufeli et al., 2002).

Social engagement examines the links of belongingness shaped between students and their classmates, educational staff, and other pertinent facts in their tertiary practice (Pekrun and Linnenbrink-Garcia, 2012). It engenders feelings of determination, socialization, and association to the tertiary source (Eldegwy et al., 2018) that are noticeable in language learning contexts through collaboration with speakers (Mercer, 2019). Social engagement in the classroom is operationally defined as directions of the learning setting, such as assistance, listening to others, taking part in a class on time, and preserving a sensible teacher-learner power construction (Pekrun and Linnenbrink-Garcia, 2012), while out of the class, it is presented through students’ involvement in groups, where ties are molded with others founded on shared principles, wellbeing, or perseverance (Wentzel, 2012). Students without social engagement are ready to undergo isolation and loneliness bringing about condensed wellbeing (MacIntyre et al., 2018).

Cognitive engagement talks about active mental states and goal-oriented learning strategies that students employ in educational activities during learning developments (Lei et al., 2018). Learners who are cognitively involved reveal a better understanding of educational work through their opinions, theories, and approaches implemented during educational activities (Khademi Ashkzari et al., 2018).

Agentic engagement refers to the learners’ participation in the current teaching that is thoughtful and originated by the student (Reeve, 2013) and this dimension of engagement is close to the other three, as it is also a practical student-originated route to educational development.

## Teachers’ Positive Behaviors

### Teacher Caring Behavior

Caring is a passion, a connection, and a behavioral sign that can be theorized as an emotion, an inspiration, and/or behavior, displaying an apprehension about other individual’s emotional states and desires (Mayseless, 2015). Teacher care refers to teachers’ performances to fulfill students’ spiritual and passionate desires through running a positive, caring, and nurturing setting (Laletas and Reupert, 2016). In the educational setting, teacher care represents a noteworthy facet of teacher-student interpersonal relationships (Gasser et al., 2018) and teacher care affects teachers’ establishment of support to students, demonstrating awareness in students’ learning (Gabryś-Barker, 2016). Caring was hypothesized to be an essential component for generating and preserving influential teacher-student interactions (Noddings, 2006), permitting teachers to concede and react to their students’ desires and provide them with safety and care (Mayseless, 2015). It has been proposed that caring is advantageous for both the care receiver and the care provider, as it stimulates the care provider’s joy, pleasure, self-assessment, social relations, and ties between them (Lavy and Bocker, 2018). In fact, teachers can stimulate the positive emotions of learners by involving them in meaningful tasks, providing a milieu that boosts their contribution in classroom negotiations, and presenting empathy (Gedzune, 2015). Some issues related to students, such as engagement, self-confidence, wellbeing, and achievement, are encouraged by teacher care (Derakhshan et al., 2019; Lavy and Naama-Ghanayim, 2020).

### Teacher Praise

Baumeister et al. (1990) as cited in Abbasi et al. (2015) declared that praise is *encouraging interpersonal feedback*. Positive feedback is categorized as a dynamic element in nurturing learners’ educational success and strengthening the preferred classroom behavior. Nicols (1995) as cited in Abbasi et al. (2015) pinpointed that positive feedback is perceived as an attractive corresponding that is in line with the learner’s self-image. Praise is generally regarded as positive feedback since it has the same meaning as it makes students feeling reinforced and meaningful and praise can be universal or specific (Moffat, 2011). The former refers to as behavior-specific praise (Hawkins and Heflin, 2011), while the latter type of praise is a well-organized and constructive educational tactic that can surge an extensive range of proper behaviors (Jenkins et al., 2015). Teacher praise is a manifestation of support or appreciation that goes further than feedback for an accurate reaction (Reinke et al., 2007). Teacher praise is regarded as a classroom strategy as dependent or a result of suitable student behaviors. In academic settings, praise should be associated with the performances or skills that the teacher desires to develop (Partin et al., 2009). Praise makes the students feel respectable, and it increases student-teacher relations through constructing a positive learning setting, diminishes troubles in the classroom, and makes learning promising (Rathel et al., 2014). To develop student engagement and success, teachers use praise regularly to reassure suitable behavior, while it reduces problematic behaviors in the classroom (Reinke et al., 2007).

## Empirical Studies

The teacher-student interactions, which occur through supportive and approachable relationships in addition to positive and promising behaviors of teachers toward students, affect foreign language satisfaction (Pishghadam et al., 2021). As stated by Mercer and Dörnyei (2020), and Li et al. (2018), teachers and some of their features, such as care, respect, helpfulness, and positive attitude, seem to be among the factors that play a prominent role in foreign language interest. When the positive teacher-student interactions are shaped, learners’ motivation, learning achievement, and engagement are developed (Henry and Thorsen, 2018). The results of the study by Royer et al. (2019) about the role of teacher praise in educational situations proved the declines of unsuitable behaviors. Teacher praise is spontaneously associated with the eminence of the student-teacher relations as Cook et al. (2018) indicated that providing praise to students can support the improvement of positive relations with learners. In the same vein, Epstein et al. (2008) acknowledged teacher praise as one of the operational approaches that support student performance and involvement and consequently undergo social and behavioral accomplishment. Rahimi and Karkami (2015) stated that teachers commonly use reward strategies in general and praise, in particular, to elude reprimand and violence strategies since these types of strategies have a destructive impact on their motivation and commitment. The study conducted by Awang et al. (2013) indicated that teachers praise is a common management strategy that is used in the classroom to manage behavior and upsurge student learning engagement in the classroom.

While there are quite a few empirical researches on Positive Psychology worldwide, there is a relatively small number of researches on Positive Psychology in China. Li (2020) has hosted a column in *Journal of the Foreign Language World*, one of the top linguistic journals in China, featuring the study of emotions in SLA. In this column, Dewaele and Li (2020) contributed a critical review on the previous theories and practices in emotion studies in SLA, proposing that the future of emotion studies can be combined with the control-value theory from the perspective of educational Positive Psychology. The next three empirical studies in the column echo with this proposal. Han and Xu (2020) investigated cases of EFL college students’ academic emotions after receiving written corrective feedback in learning second language writing and their emotion-oriented‚appraisal-oriented‚and situation-oriented self-regulation strategies. The study has generated implications for using Positive Psychology to facilitate students’ wellbeing. Another empirical study conducted by Jiang (2020) used focused essay technique to examine teacher-related factors in affecting EFL classroom enjoyment. Jiang’s research has implications for promoting Positive Psychology in China’s EFL classrooms. The last empirical research article by Li (2020) is also conducted in the realm of Positive Psychology. The method of questionnaire and self-rating test is used to investigate students’ emotional intelligence, emotions, and their relationship with English achievement. Emotions, such as enjoyment, anxiety, and burnout, exert influence on students’ emotional intelligence in general. Emotions and EI have correlations with English achievement. Li’s (2020) research offers a unique perspective of understanding emotion intervention in L2 pedagogy. This column has demonstrated a variety of research methodologies and perspectives, covering a wide range of emotions in EFL learning, such as enjoyment and emotional intelligence, and has implications for using Positive Psychology in EFL setting.

Li’s research interest remains in Positive Psychology of SLA. Li (2021) critically reviewed the researches in PP from the past to the present, advocating that the conception of positive language education will promote language learning emotions as well as the wellbeing of the students. Positive Psychology in SLA has attracted research interest not only in China, but also in worldwide. Empirical studies are still in urgent need to investigate the role of PP in language teaching and learning.

## Implications and Future Directions

The current review may have some implications for researchers, teachers, and teacher educators in the EFL context. From the operational viewpoint, developing the interrelations of teacher and student features in the EFL milieu can be significant in developing learner engagement. Likewise, this study can be of help to the language teacher in search of ways to improve student engagement.

This review can help teachers to be acquainted with the status of some individual aspects like teacher caring behavior and praise in inspiring positive results concerning student engagement. Thus, teachers and teacher trainers keen on increasing the positive upshots of EFL classes can meditate on the findings of this study and make enhancements in their works. Based on the low degrees of learner engagement, it is necessary for teachers to scrutinize methods to enthusiastically involve learners in the classroom (Nguyen et al., 2018). Teachers evinced that distractions, rebellion, and disengagement are among the most reliably demanding and unsatisfying manners with which they face in their life (Alter et al., 2013). Learner engagement is promoted and nurtured with the mastery of the use of motivating teaching behaviors (Nicholson and Putwain, 2018). More precisely, the teaching behaviors should embrace implementing great levels of relatedness care from the outset and teachers need to engross learners in the syllabus with learning tasks, while witnessing the learners with endurance and providing regulation with positive feedback, namely, praise and support during interventions. Based on the literature review, the authors concluded that teachers’ classroom management and learner engagement, and the relations between learner and teacher are significantly integrated.

Teacher praise is a widespread classroom-managing approach that successfully impacts student learning (Floress et al., 2017). Praise is an active and concrete policy that is employed to surge learners’ prosocial behaviors (Dufrene et al., 2014). Teacher praise and overt inspiration can also be salient for constructing students’ self-confidence (Dweck, 2007). Assisting teachers to utilize praise in the classrooms is prominent as it can prevent student problems. However, the fact that several teachers do not logically arranged for more positive than negative in their classrooms (Reinke et al., 2013; Derakhshan et al., 2021) specifies that it is required to conduct further study to investigate what teaching approaches and types of praise will result in successful teacher praise. Undoubtedly, having a more detailed perception of how praise functions in EFL classrooms will assist all learners to be efficacious in the EFL classroom. Although the significant effect of teacher praise is certified in language learning, studying the kinds of praise can be most efficient will be worthwhile in evolving proper proficient progress for teachers in further studies.

Teachers can encourage positive relations through activities that convey hope, compassion, and care and in which they identify the independence and individuality of students (Gkonou and Mercer, 2018). All learners have strong points, and it can be essential for them to be able to ascertain these and use them (Mercer, 2019). Teacher care speaks of teacher-originated actions that cultivate positive social ties with learners which demands sustaining a classroom setting in which the learners feel appreciated and are simultaneously respectful of the teacher as the power character (Ware, 2006). The caring relation constructed between teacher and student through interactions appeared to manage their conception and creations of themselves. When learners feel that the teacher is caring for them, they become more self-confident and regard themselves as superior learners. Sequentially, for the teachers, students’ mutuality permitted them to perceive the positive effect of their caring for most learners leading to their engagement in the process of learning. Teacher caring behavior has been related to a widespread series of positive results comprising higher presence, enhanced academic success, and decreased drop-out proportion (Foster, 2008).

Additionally, material developers can take advantage of this route of study by considering them when adjusting teacher and learner books. In the same vein, syllabus designers are supposed to reflect effective teacher-student relations as a basis of learning to design tasks and activities in a way to uphold teacher-student negotiations and interactions to undertake tasks and engage them. However, there is an absence of inquiries on how teacher’s positive behaviors, such as teacher praise and caring behavior, can be enhanced in the training courses to be efficient in engaging learners and further consideration should be given to different dimensions of engagement.

## Author Contributions

The author confirms being the sole contributor of this work and has approved it for publication.

## Funding

This study is sponsored by the China Postdoctoral Science Foundation (Project No. 2018M642877) and MOE (Ministry of Education in China) Project of Humanities and Social Sciences (Project No. 18YJC740088).

## Conflict of Interest

The author declares that the research was conducted in the absence of any commercial or financial relationships that could be construed as a potential conflict of interest.

## Publisher’s Note

All claims expressed in this article are solely those of the authors and do not necessarily represent those of their affiliated organizations, or those of the publisher, the editors and the reviewers. Any product that may be evaluated in this article, or claim that may be made by its manufacturer, is not guaranteed or endorsed by the publisher.
